# Targeting FAK, VEGF, and MTA1 proteins with *Terminalia elliptica*: a computational approach for anticancer activity

**DOI:** 10.3389/fonc.2024.1427632

**Published:** 2024-09-17

**Authors:** Bhargav Shreevatsa, Shrivatsa Hegde, Prakruthi Narayan, Chandan Dharmashekar, Anisha Jain, Tanveer A. Wani, Samudyata C. Prabhuswamimath, Shiva Prasad Kollur, Chandan Shivamallu

**Affiliations:** ^1^ Department of Biotechnology and Bioinformatics, JSS Academy of Higher Education and Research, Mysuru, Karnataka, India; ^2^ Department of Microbiology, JSS Academy of Higher Education and Research, Mysuru, Karnataka, India; ^3^ Department of Pharmaceutical Chemistry, College of Pharmacy, King Saud University, Riyadh, Saudi Arabia; ^4^ School of Physical Sciences, Amrita Vishwa Vidyapeetham, Mysuru, Karnataka, India

**Keywords:** FAK, VEGF, MTA1, phytobioactives, *Terminalia elliptica*

## Abstract

Cancer remains a significant global health challenge, prompting exploration into alternative treatments such as those derived from natural compounds found in traditional medicine. Recent research has underscored the role of proteins like Focal Adhesion Kinase (FAK), Vascular Endothelial Growth Factor (VEGF), and Metastasis-Associated Protein 1 (MTA1) in driving cancer cell proliferation and survival. Here, we investigated the potential of a single molecule to modulate these key proteins involved in metastasis, offering a promising avenue for cancer therapy. Terminalia elliptica, commonly known as Asna, possesses a diverse range of medicinal properties, including antimicrobial, anti-inflammatory, anticancer, antidiabetic, antiaging, hepatoprotective, antioxidant, and neuroprotective activities. Our study aimed to explore the anticancer potential of *Terminalia elliptica* by identifying bioactive compounds capable of targeting FAK, VEGF, and MTA1 to impede cancer metastasis. Through in silico analysis, we conducted network analysis using Cytoscape to assess the significance of these bioactive compounds in the inhibition of signaling pathways driving metastasis. The utilization of these bioactives as potential candidates for targeted therapy of VEGF, FAK, and MTA1 regulated pathways was preliminarily assessed by Molecular Docking and MD Simulation. Our findings revealed that phytobioactives namely, Chebulinic Acid of *Terminalia elliptica*, exhibited notable binding affinity and interaction with FAK, and Chebulagic Acid with VEGF, and MTA1. This discovery holds promise as a novel therapeutic approach for combating cancer, offering potential benefits in cancer treatment and management.

## 1 Introduction

**Figure 1:**
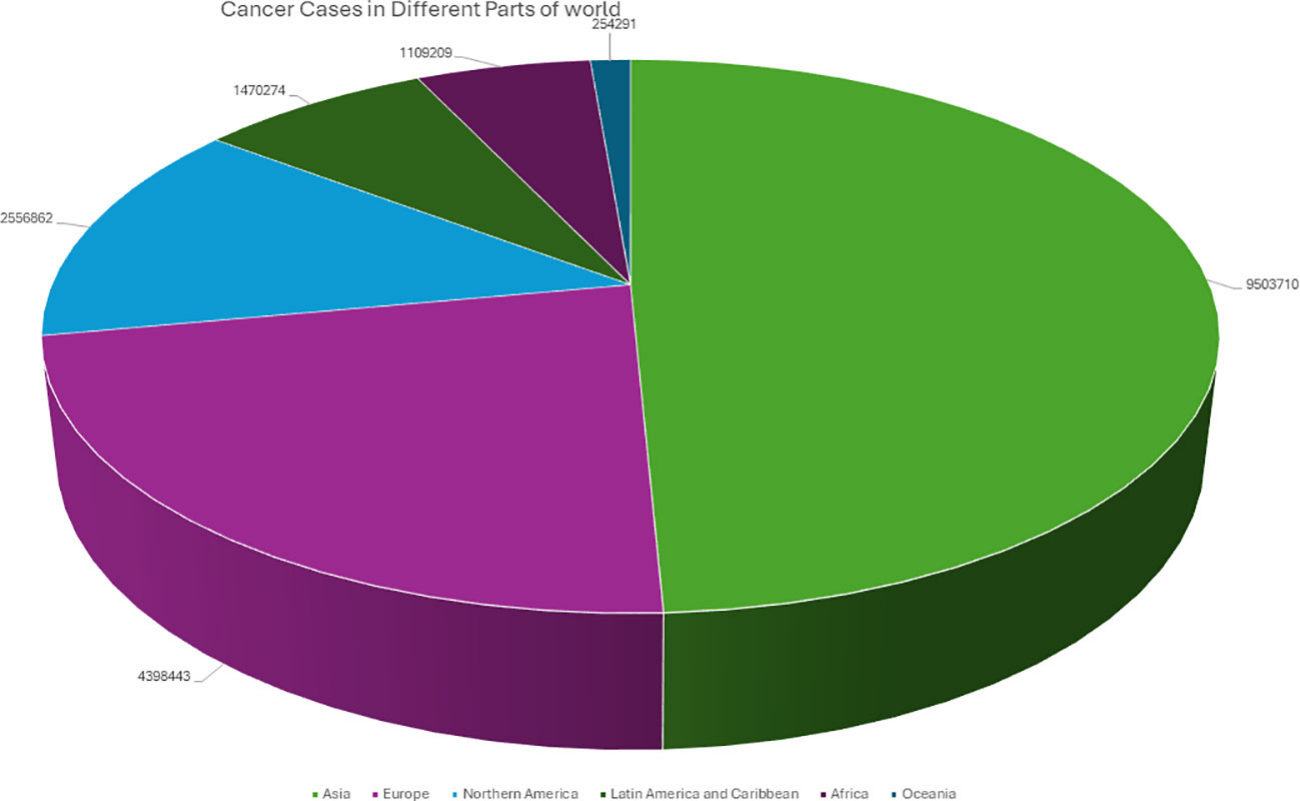
Graphical representation of the Number of cancer patients from different parts of the world in 2020.

**Figure 2:**
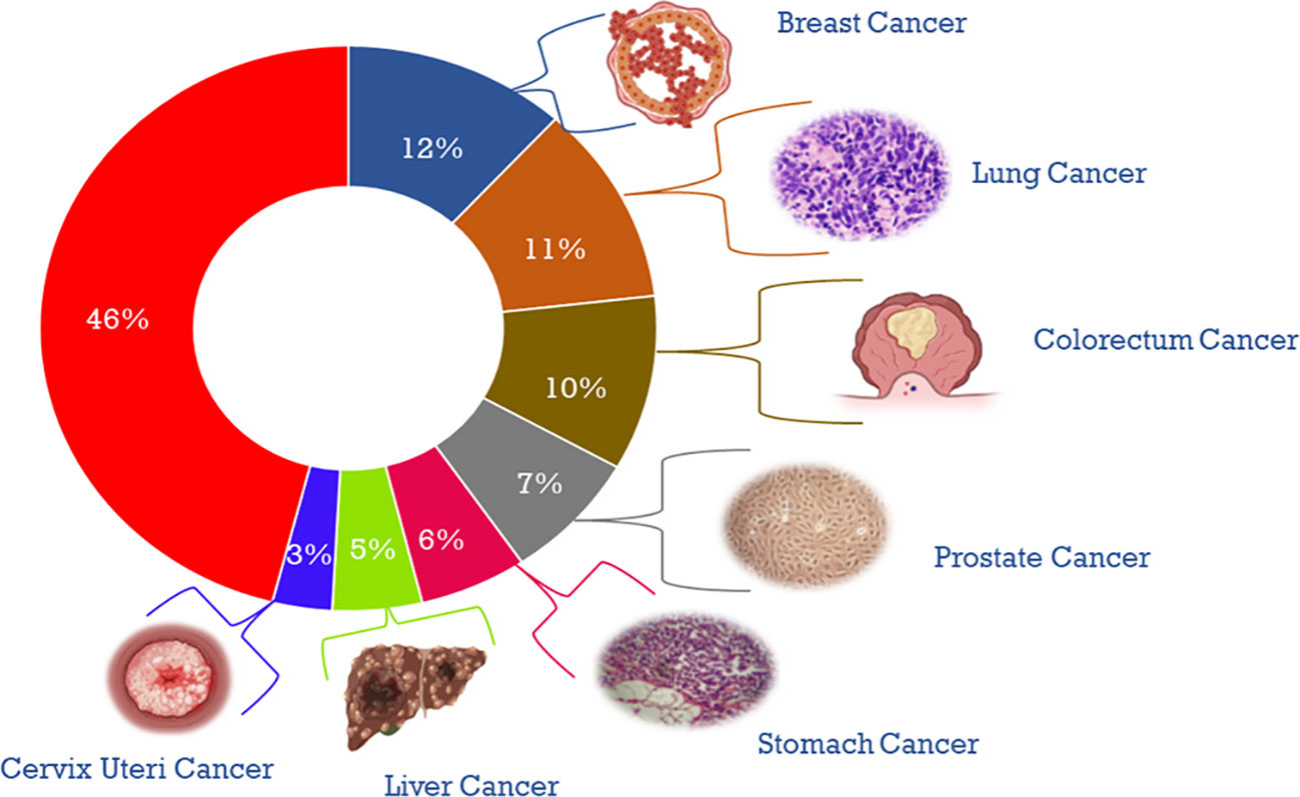
Diagrammatical representation of the percentage of cases of different cancers in 2020.

Cancer is a complex disease that arises from the uncontrolled growth and division of abnormal cells, often resulting in tumor formation (1). It continues to pose significant challenges to medical researchers and clinicians (2). According to the World Health Organization, the global burden of invasive cancer in 2020 is staggering, with an estimated 2.3 million new cases in both sexes combined, and 685,000 deaths, primarily among women (3). The main objective of this initiative is to decrease cancer mortality rates by promoting public awareness regarding cancer risk factors and symptoms. Representing a quarter of all cancer cases in females, breast cancer is the most diagnosed cancer among women, and the burden of the disease has been growing in many parts of the world, particularly in transitioning countries (**Figure 1**) (4). Cancer is also a significant public health concern in India, with an estimated 100,000 new cases reported annually and a case-fatality ratio of 40% (5). This makes India one of the countries with the highest number of cancer-related deaths (6). I the percentage of cases of different cancers in 2020 (**Figure 2**). In response to this crisis, the World Health Organization launched the Global Cancer Initiative to address this public health concern, which can lead to early detection, timely diagnosis, appropriate treatment, and effective patient management (7). With a better understanding of global patterns and variations in disease burden, the initiative hopes to improve outcomes for those affected by cancer.

Cancer management and treatment with conventional approaches of chemotherapy has become a major challenge due the problem of side effects and chemoresistance. Also, the ever-evolving hallmarks of cancer progression like self-sufficiency to growth signals, insensitivity to growth suppressor signals, immune evasion and apoptosis evasion, enabling replicative immortality, sustained angiogenesis, invasion and metastasis, acquisition of phenotypic plasticity, non-mutational epigenetic reprogramming and the contribution of microbiome in tumor progression has made the adaptation of multimodal approaches inevitable for better therapeutic outcomes. Hence the investigation of natural molecules for targeting multiple proteins could be an effective approach for improved treatment outcomes. Cancer metastasis, which refers to the migration of cancer cells from their original location to distant organs, plays a significant role in cancer-related death (8). The metastatic process is multifaceted and involves various steps and the participation of numerous proteins such as matrix metalloproteinases (MMPs), epithelial-mesenchymal transition (EMT), CCL2, CXCL12, and CXCL16, as well as their corresponding receptors (9–12). These proteins are part of the normal cell cycle and contribute to the complex mechanisms of metastasis (13). Proteins, such as MTA1, play a significant role in regulating gene expression and facilitating cancer cell invasion and migration. MTA1 modulates the expression of genes related to epithelial-mesenchymal transition (EMT), a process by which cancer cells acquire a more invasive and migratory phenotype. Additionally, MTA1 is also involved in regulating the expression of matrix metalloproteinases (MMPs), which are enzymes that facilitate cancer cell invasion and migration by degrading the extracellular matrix. Another mechanism of cancer progression is angiogenesis, which is the formation of new blood vessels from existing ones and is essential for tissue repair. However, in conditions such as cancer, angiogenesis plays a crucial role in tumor growth and progression. Two proteins, namely VEGF and FAK, have been found to participate in various stages of angiogenesis. VEGF and FAK are involved in several signaling pathways that regulate angiogenesis, including the PI3K/Akt, MAPK/ERK, and JAK/STAT pathways (14–17). VEGF primarily activates the PI3K/Akt and MAPK/ERK pathways, while FAK regulates the JAK/STAT pathway, highlighting its crucial roles in the development of new blood vessels and their potential as therapeutic targets for cancer treatment (18, 19). Targeting these key proteins, MTA1, VEGF, and FAK, can significantly affect the pathways and underlying mechanisms contributing to cancer development and progression (20, 21).

**Figure 3:**
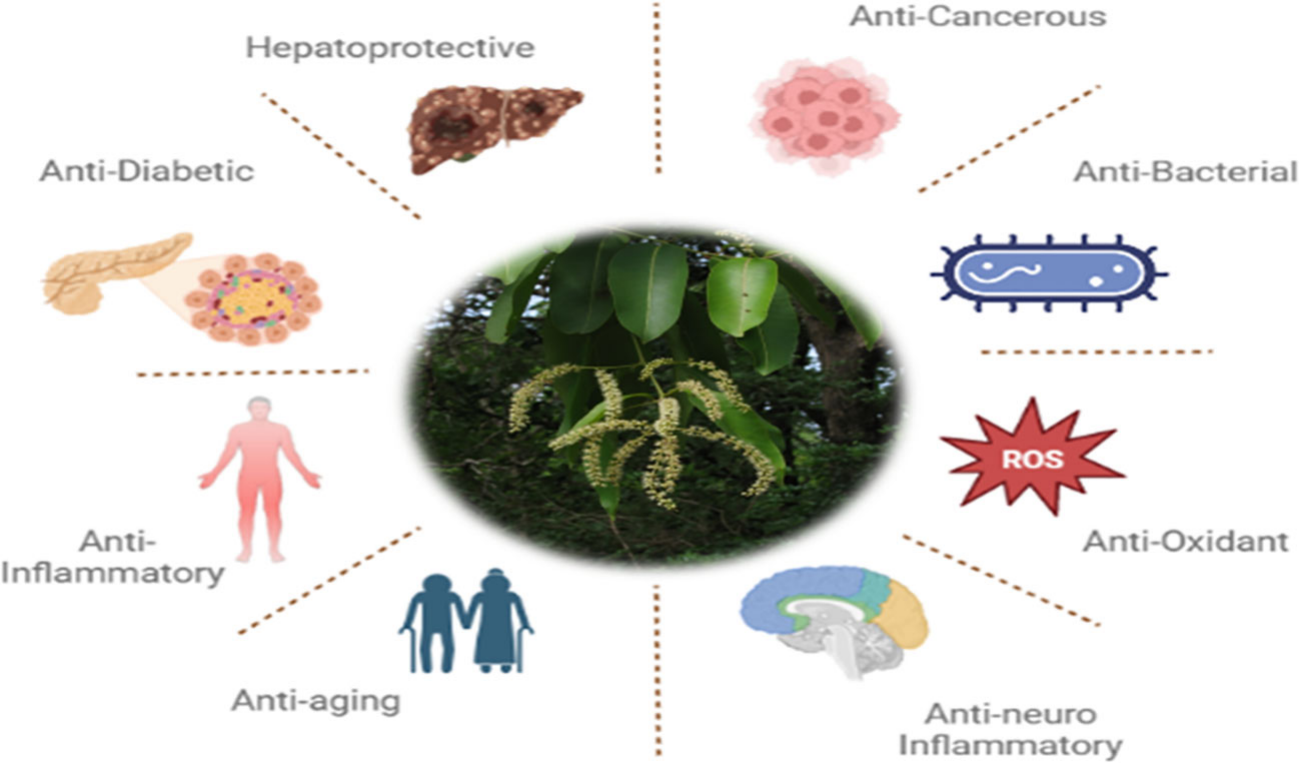
Diagrammatical representation of medicinal properties of *Terminalia elliptica*.

The *Terminalia elliptica* plant, also called Asna, exhibits various medicinal properties such as anti-microbial, anti-inflammatory, anti-cancer, anti-diabetic, anti-aging, hepato-protective, antioxidant, and neuroprotective activities (**Figure 3**) (22–24). This evidence suggests we investigate the potential anti-cancer activity of Terminalia Elliptica. The current in silico study focuses on identifying and inhibiting the proteins that are commonly associated with Cancer.

As the tumor progression and metastasis is regulated by angiogenic pathways, wherein VEGF plays a predominant role and is the most abundantly secreted proangiogenic growth factor. The MTA1 protein (Metastasis Associated Protein1) regulates tumor progression and metastasis by interaction with multiples genes and protein targets that control tumor cell transformation, angiogenesis, invasion, metastasis and resistance to therapy, and is hence regarded as the ‘hub’ of cancer. Focal adhesion kinase (FAK), a non-receptor tyrosine kinase and an adaptor protein is crucial for signaling mechanisms that regulate cell adhesion and cell migration (25). It is overexpressed in cancerous conditions and is considered as a highly druggable target, that when used especially in combinatorial therapies can reverse chemotherapeutic resistance or targeted therapy failures and enhance immune-oncological based treatments. Considering the crucial functions of these three key players in modulation of tumor proliferation, angiogenesis and metastasis, we have strategized a novel multiprotein targeted approach using natural molecule by targeting FAK, VEGF, and MTA 1 with *Terminalia elliptica* using computational approaches.

## 2 Materials and methods

### 2.1 Identification and acquisition of phytobioactives

A comprehensive list of 90 phytobioactives found in the leaves of the *Terminalia elliptica* plant was compiled and used as the basis for the research shown in **Table 1** (26).

**Table 1:** List of major phytobioactives present in *Terminalia elliptica* leaves.

Compound name	CID ID	Molecular Formula	Molecular weight (g/mol)
Amentoflavone	5281600	C_30_H_18_O_10_	538.5
Arjunetin	21152828	C_36_H_58_O_10_	650.8
Chebulagic acid	250397	C_41_H_30_O_27_	954.7
Chebulinic acid	72284	C41H32O27	956.7
Isovitexin	162350	C_21_H_20_O_10_	432.4
Orientin	5281675	C_21_H_20_O_11_	448.4
Rutin	5280805	C_27_H_30_O_16_	610.5
Naringin	442428	C_27_H_32_O_14_	580.5

### 2.2 Preparation of ligands

The chosen ligands were obtained from the USA-PubChem Chemistry Database in their three-dimensional form represented in **Table 2**. The geometric properties of the ligands were then optimized using Argus Lab version 4.0.1 [http://www.arguslab.com/, (accessed on 16 September 2022)] (27).

**Table 2:** 2D and 3D structure of major phytobioactives present in *Terminalia elliptica* leaves.

Compound name	Molecular weight (g/mol)	2D structure	3D structure
Chebulinic Acid	956.7	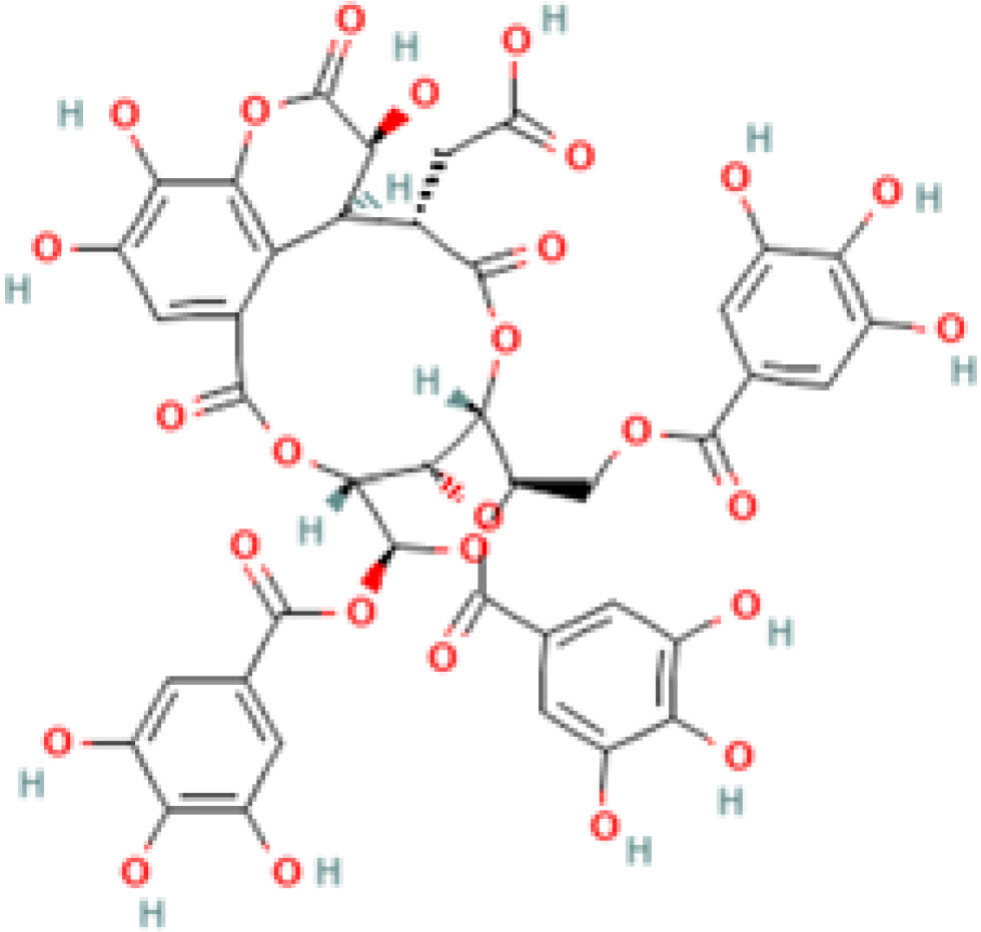	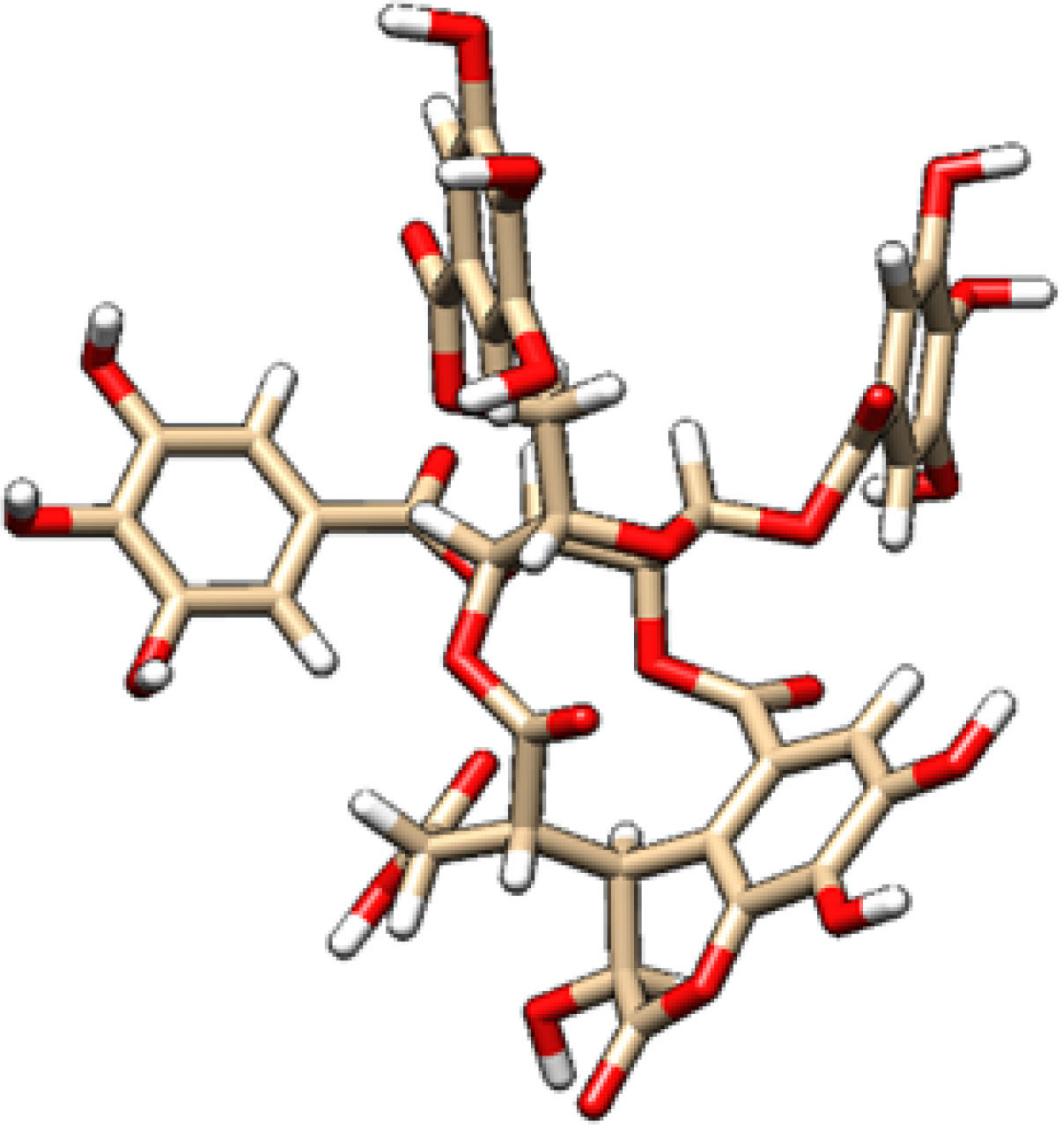
Chebulagic Acid	954.7	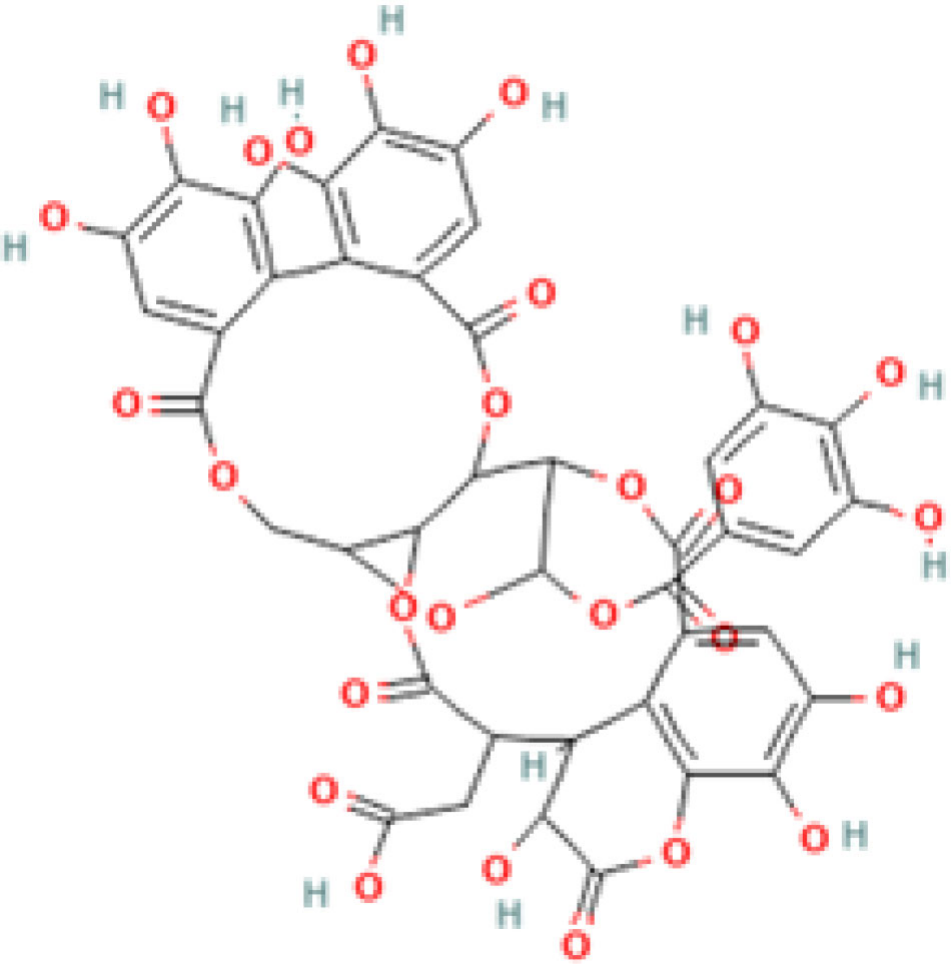	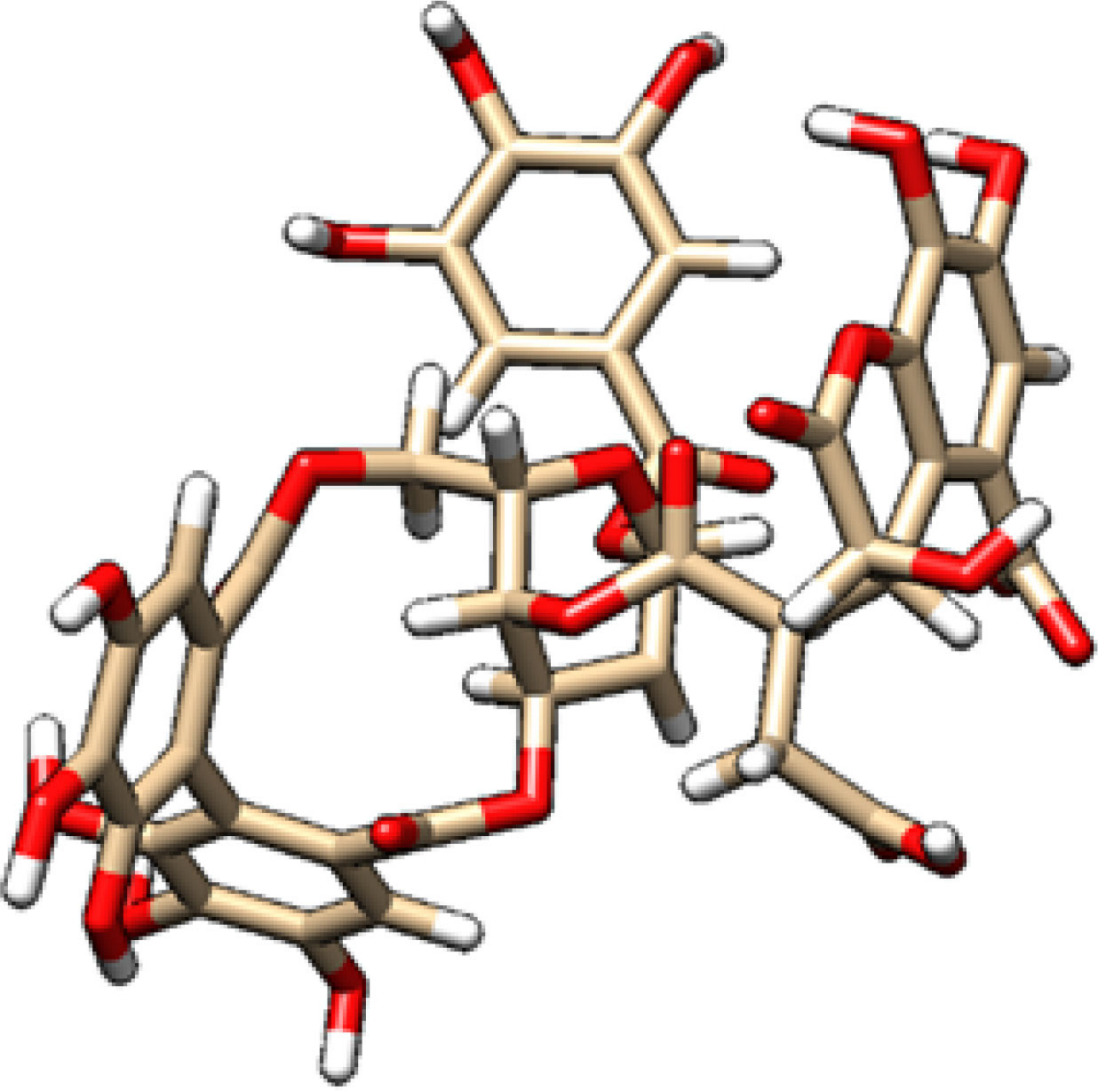
Isovitexin	432.4	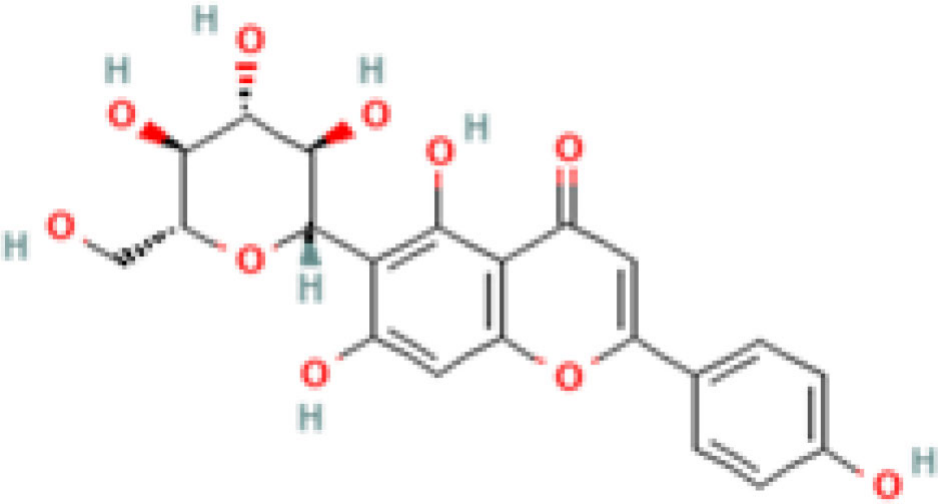	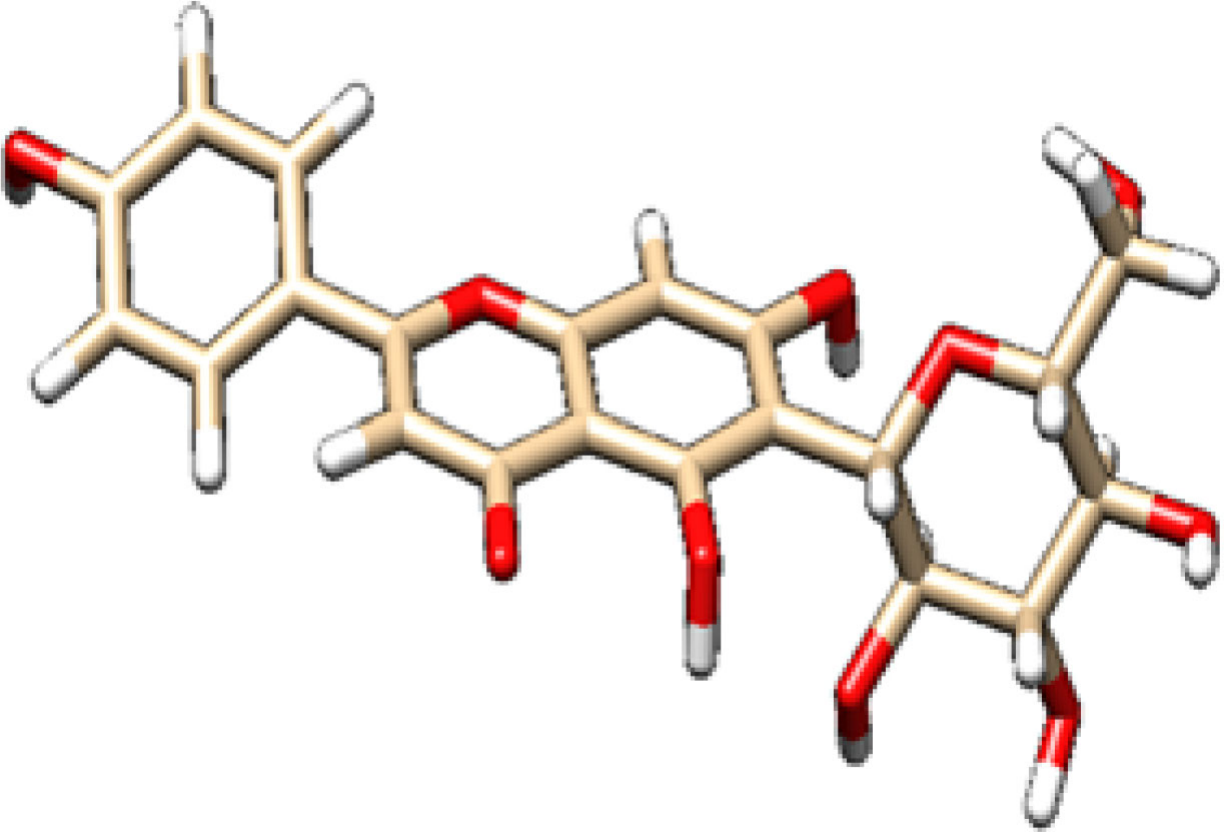
Naringin	580.5	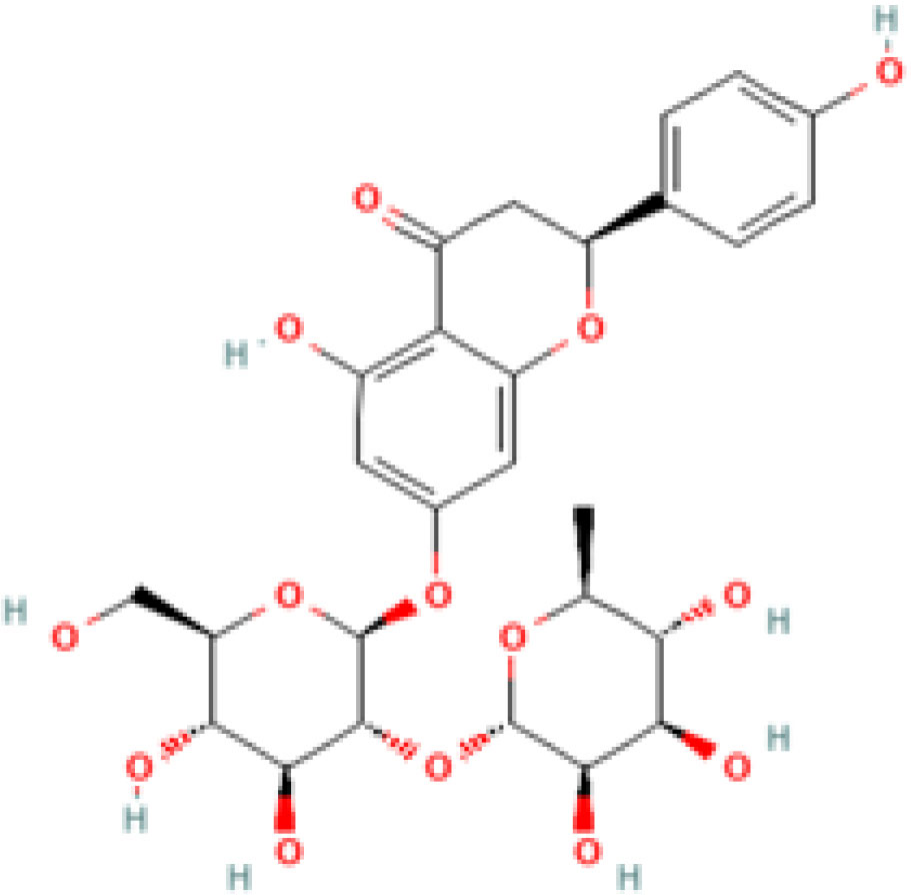	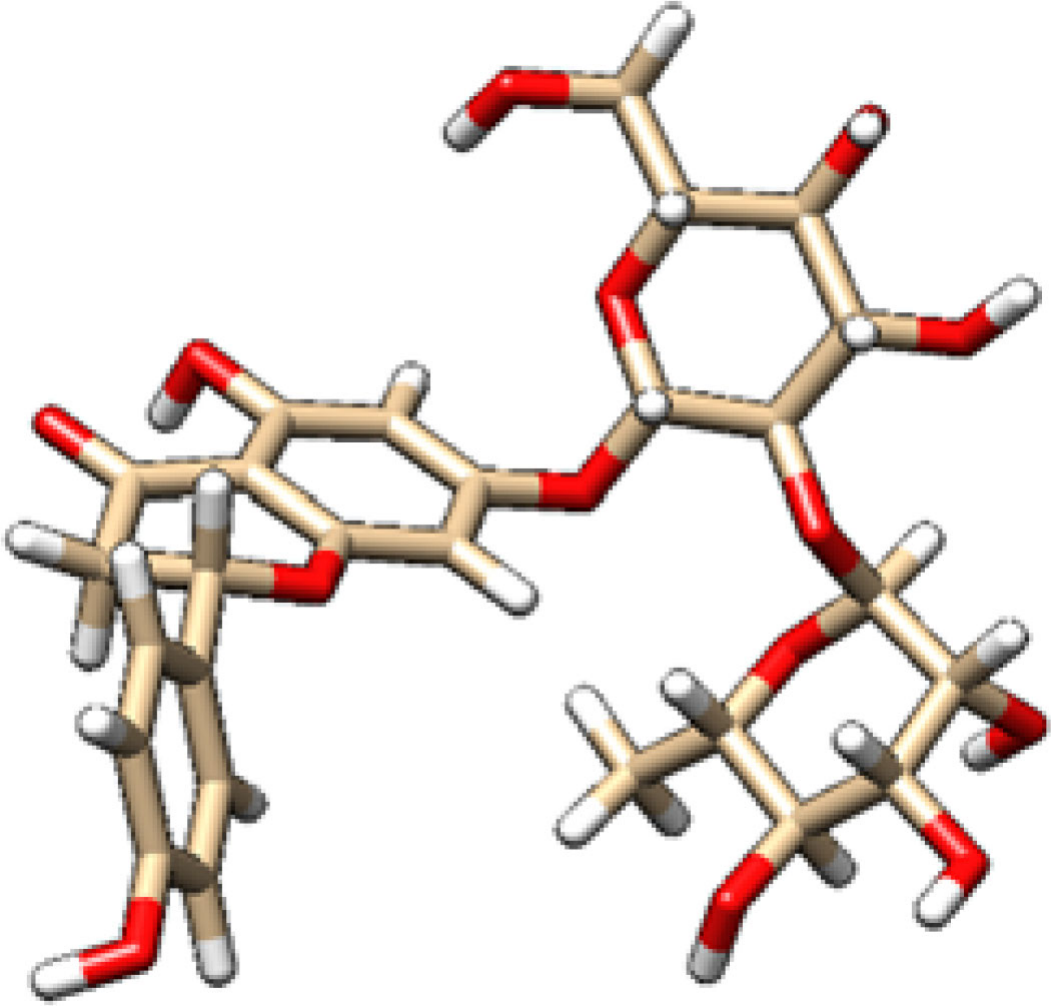

### 2.3 Construction of PPI network

To explore the interactions and associations among disease-related genes, we uploaded them to the STRING database (https://string-db.org/), a comprehensive online resource for protein-protein interactions in molecular biology. The resulting PPI network was then imported into Cytoscape, an advanced and open-source software, for further analysis and visualization (28).

### 2.4 Protein preparation

The three-dimensional crystal structure of the Focal Adhesion Kinase (FAK) protein (PDB ID:1MP8), VEGF and MTA1 protein (PDB ID: 4BKX) was obtained from the Protein Data Bank (PDB). Pymol software was used to remove or modify any non-standard amino acids present in the downloaded FAK and MTA1 PDB file. Additionally, missing residues were fixed using MODELLER 10.3 software (https://salilab.org/modeller/) (29, 30).

### 2.5 Binding site prediction

The potential binding sites of the proteins were predicted using the *In silico* tool CASTp (http://sts.bioe.uic.edu/castp/index.html?3trg) on the refined structure obtained from MODELLER (29).

### 2.6 Protein structure validation

The refined FAK, VEGF and MTA1 structure was validated using the Ramachandran plot generated by the PROCHECK RAMPAGE option in UCLA-DOE LAB-SAVES v6.0 (https://saves.mbi.ucla.edu/). The results indicate a stable and well-validated structure (31).

### 2.7 Molecular docking analysis

Molecular docking studies were conducted using Pyrx version 0.8 open-access docking program to understand the interactions between proteins and the phytobioactives. The proteins in PDB format and the ligands were loaded, and the active site residues were marked to define the grid box. The highest orientation with the lowest binding affinity (Kcal/mol) values were obtained (27, 29).

### 2.8 Molecular docking visualization

The docked conformation of the ligands against the proteins were visualized using BIOVIA Discovery Studio Visualizer. This program was used to create 3D and 2D visualizations of the interactions between protein and several plant-derived compounds, including hydrogen bonds, hydrophobic interactions, and bond length (27, 29, 31).

### 2.9 Molecular dynamic simulation

A Molecular Dynamics Simulation (MDS) was conducted using Desmond module of Schrodinger suite 2022-3 version. The MDSs for Chebulinic Acid-MTA1, Chebulinic Acid-VEGF and Chebulagic Acid-FAK were carried out for 100 nanoseconds. A system-builder such as TIP3P was used to insert the water model in the docked protein-ligand complex, inside an orthorhombic periodic border of the box, with the system in a solvated state. To balance the system electrically, Na+ and Cl- ions were introduced into the protein water system. This step ensures the charge of the system is neutralized when the ligand is competing at the binding site. Post completion of the simulation run, the obtained results and trajectories were analyzed using the simulation interaction diagram panel of the Schrödinger desmond module (27, 30, 31).

### 2.10 ADMET

The identification of phytobioactives from various medicinal plants was done using pharmacokinetic and toxicity (ADMET) profiles. Poor ADMET (absorption, distribution, metabolism, excretion, and toxicity) characteristics have been shown to deteriorate pharmaceutical activity. Additionally, in silico methods were used to assess the ADMET characteristics to see if the screened phytobioactives would make good candidates for appropriate medication. Practical reasons for drug failure include toxicity and undesirable pharmacokinetics. Finding this during the clinical phase is expensive. Based on AMES toxicity, the toxicity profile of all screened phytobioactives from different medicinal plants (27).

## 3 Results and discussion

### 3.1 Analysis of PPI network

**Figure 4:**
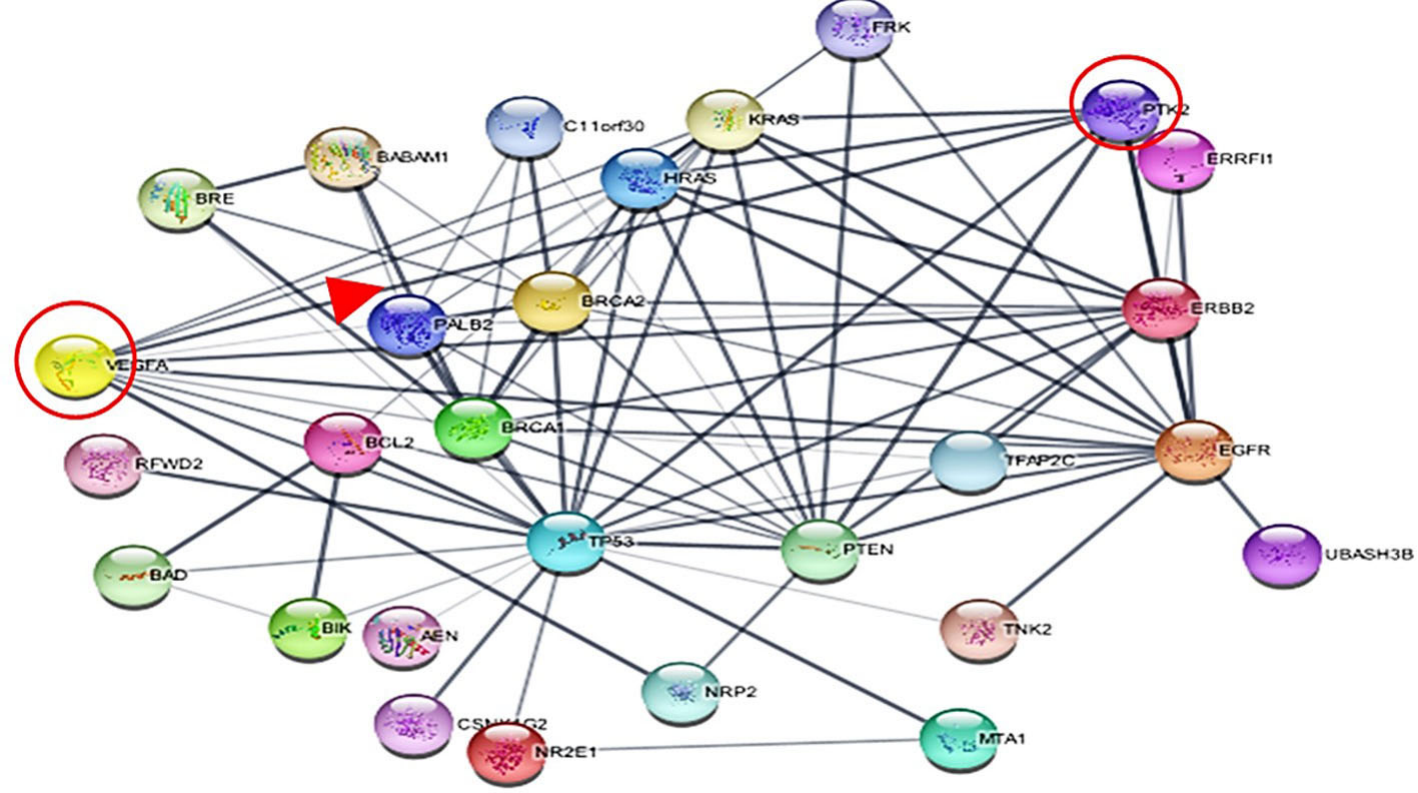
The network illustrates the interactions among the selected proteins.

We investigated the role of specific proteins in cancer by selecting important proteins involved in cancer, such as VEGF, PTK2 (FAK), and MTA1, and then uploaded to the STRING database. STRING database is an online resource that provides information on both known and predicted protein-protein interactions. The STRING database provides information from diverse sources, such as experimental data, computer prediction methods, and publicly available text collections. After importing the resulting network into Cytoscape for analysis, we identified three main proteins in the network, with two having many interactions shown in (**Figure 4**). Cytoscape is an advanced, open-source software that can be downloaded free of charge via their website (https://cytoscape.org/download.html). Our analysis of the network provided valuable insights into the importance and relationships of key proteins, which can aid in a better understanding of the underlying mechanisms of cancer and the identification of potential therapeutic targets.

### 3.2 Protein structure validation

**Figure 5:**
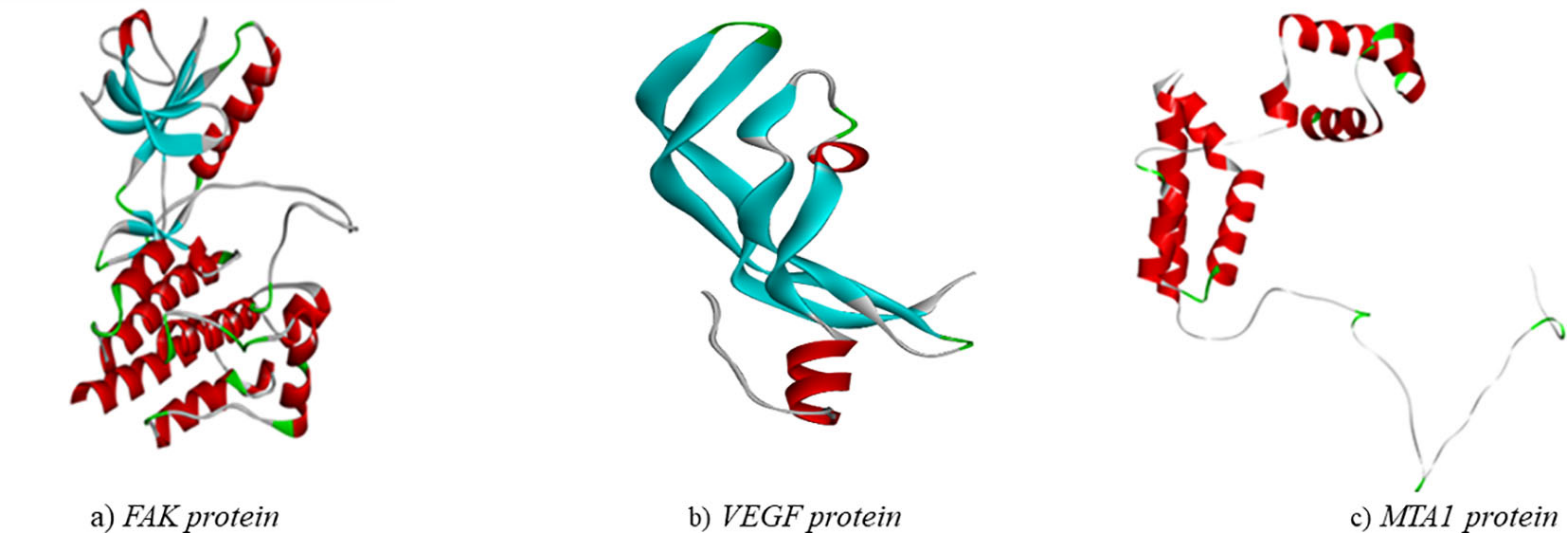
Three-dimensional structure of targeted proteins **(A)** FAK. **(B)** VEGF **(C)** MTA1.

**Figure 6:**
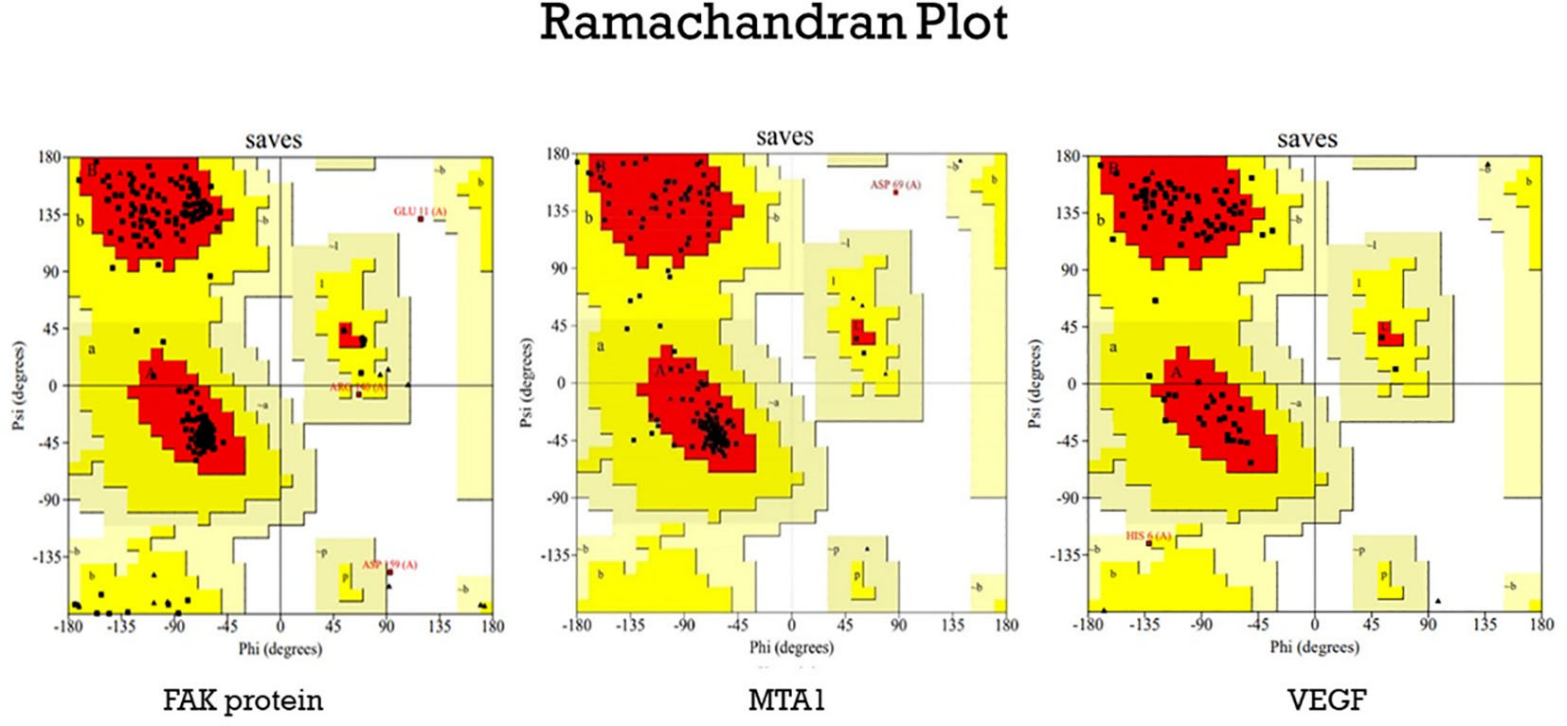
The Ramachandran plot generated from RAMPAGE. The Ramachandran plot representing energetically allowable regions for backbone dihedral angles ψ vs. φ amino acid residues in selected protein structure.

The structure of the proteins (**Figure 5**) were validated using the Ramachandran plot generated by PROCHECK RAMPAGE. The results (**Figure 6**, **Table 3**) showed that the residues were in the favorable regions, indicating that the structure was suitable for further molecular docking studies.

**Table 3:** List of proteins and their percentage of residues in favored regions.

SI. No.	Protein Structure	Number of Residues in Favored Regions (%)	Number of Residues in Allowed Regions (%)	Number of Residues in Disallowed Regions (%)
1	VEGF	91.1	7.8	0.0
2	MTA1	91.2	8.1	0.6
3	FAK	92	6.8	0.8

### 3.3 Protein-ligand interaction

**Figure 7:**
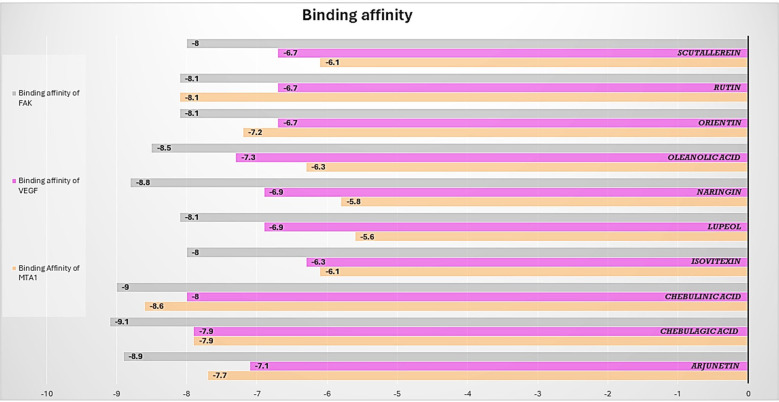
Barplot of Molecular Docking results between selected proteins against selective phytobioactives (the binding energy value δG is shown in minus kcal/mol).

**Figure 8:**
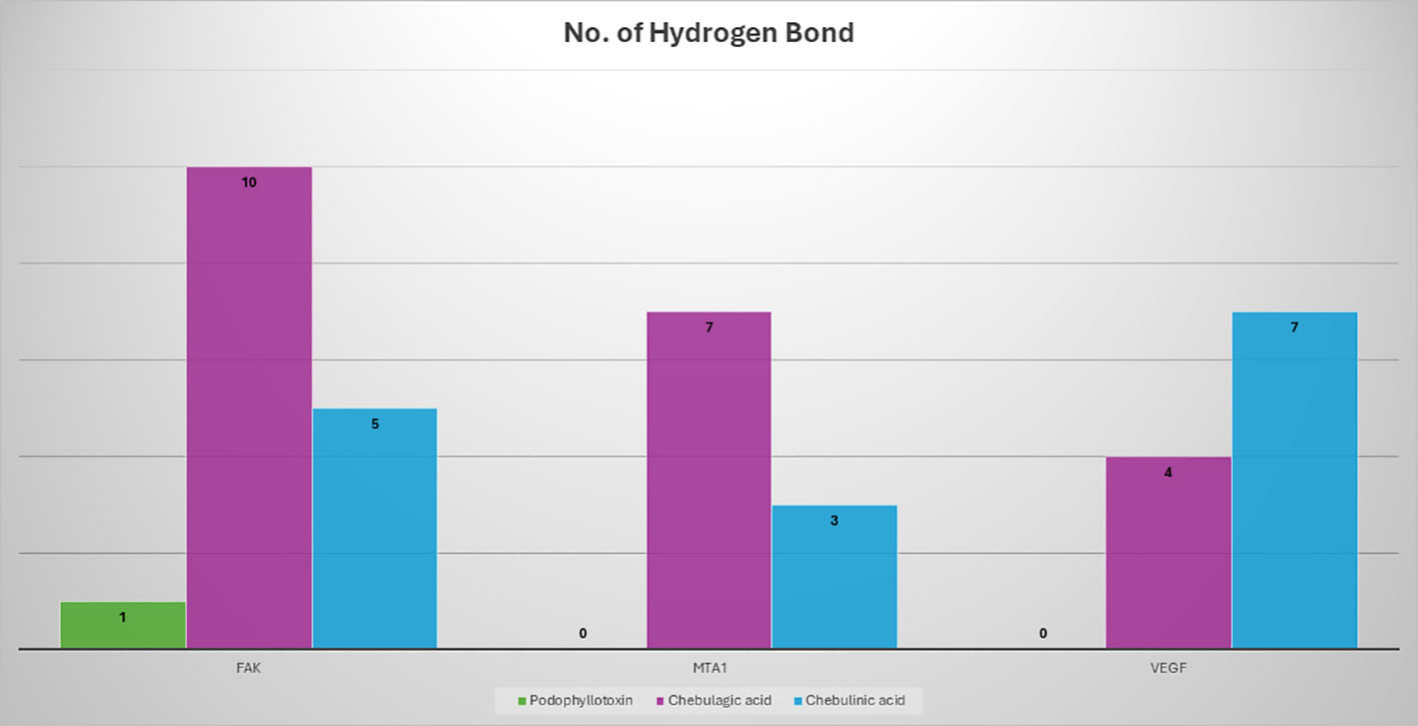
A comparison of the potency of various phytobioactives and a standard inhibitor was conducted, with the number of hydrogen interactions with proteins plotted on the y-axis and the different phytobioactives plotted on the x-axis.

The results of the molecular docking studies between the selected proteins and the phytobioactives are presented in (**Figures 7**, **8**, **Table 4**). The best-docked pose among the various compounds was selected based on the lowest binding affinity (Kcal/mol) values. The interactions between the selected ligands and the proteins were analyzed using the BIOVIA Discovery Studio Visualizer software, highlighting the key binding residues. The selected ligands were observed to form hydrogen bonds with the protein that were shorter than 2.75 nm in length and were also partially surrounded by hydrophobic interactions.

**Table 4:** Details of intermolecular interactions.

Target Proteins	Ligands	Binding Affinities (kcal/mol)	Hydrogen bond forming residues
FAK	Chebulinic Acid	-9	HIS 139 (2 bonds), ASP 159, LEU 162 (2 bonds), TYR 171 (2 bonds), GLU 167 (2 bonds), GLY 177
VEGF	Chebulinic Acid	-8	HIS 3, GLN 17 (2 bonds), CYS 21 (2 bonds), HIS 22, CYS 97
MTA1	Chebulinic Acid	-8.6	ARG 88 (2 bonds), THR 162, GLU 166 (2 bonds), TYR 169, LYS 172

### 3.4 Analysis of selected protein-phytobioactive interactions

**Figure 9:**
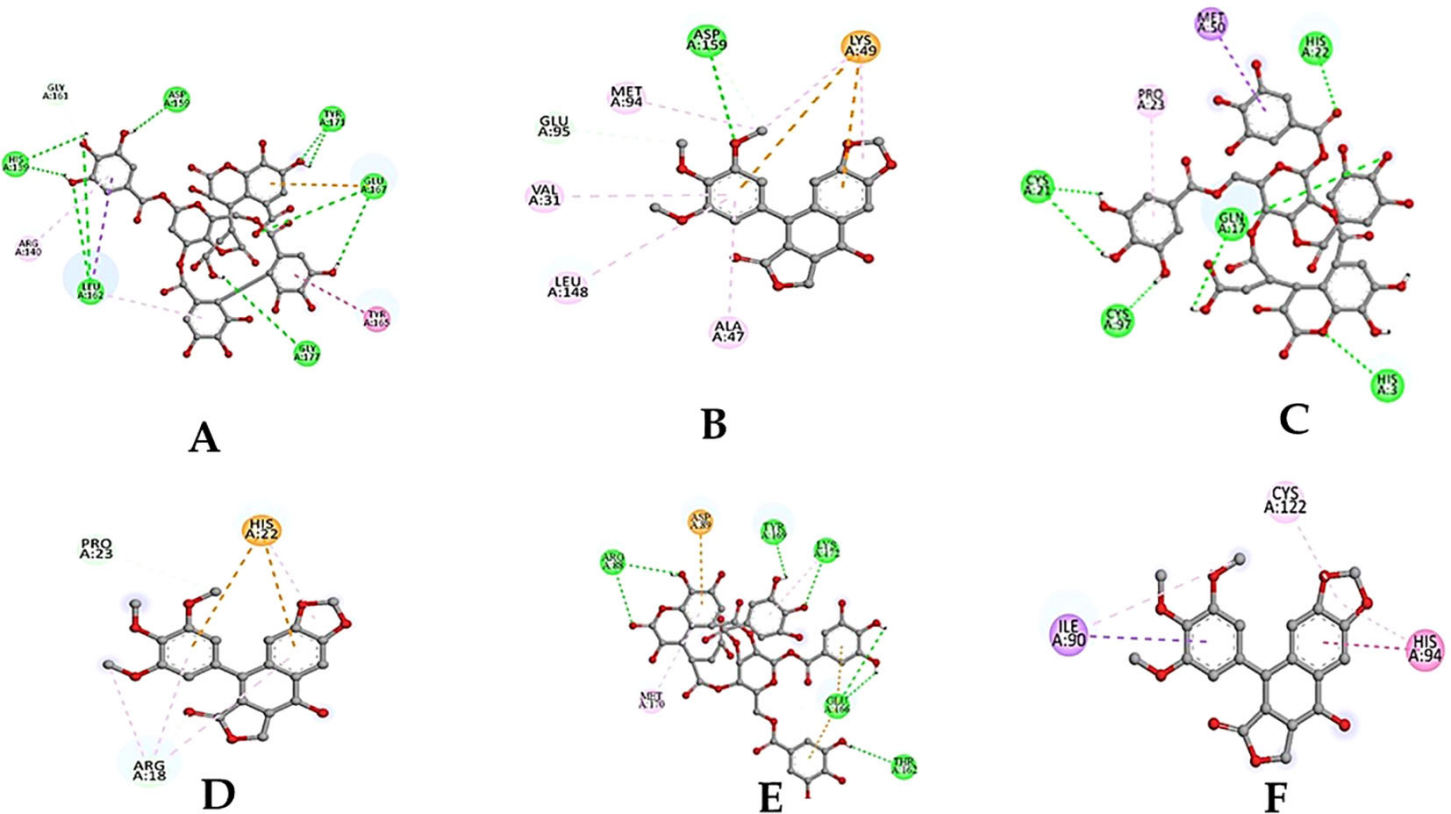
**(A)** 2D-3D structure of Chebulagic acid docked with FAK. **(B)** 2D-3D structure of standard drug docked with FAK. **(C)** 2D-3D structure of Chebulinic acid docked with VEGF. **(D)** 2D-3D structure of standard drug docked with VEGF. **(E)** 2D-3D structure of Chebulinic acid docked with MTA1. **(F)** 2D-3D structure of standard drug docked with MTA1.

The molecular docking studies between the selected protein and the selected phytobioactives revealed key interactions between the ligands and the proteins. The results, as presented in (**Figures 9A-F**), showed that chebulagic acid had the highest binding score, indicating a strong binding affinity for the FAK protein and Chebulinic acid had the highest binding score, indicating a strong binding affinity for the VEGF and MTA1 proteins. A detailed analysis of the interactions using PyMOL software revealed that chebulagic acid and chebulinic Acid formed several hydrogen bonds with key amino acid residues in the FAK protein and VEGF, MTA1 protein respectively. Our findings suggest that chebulagic acid and chebulinic acid could be a potential candidate for further studies as a regulator in cancer.

### 3.5 ADMET analysis

**Figure 10:**
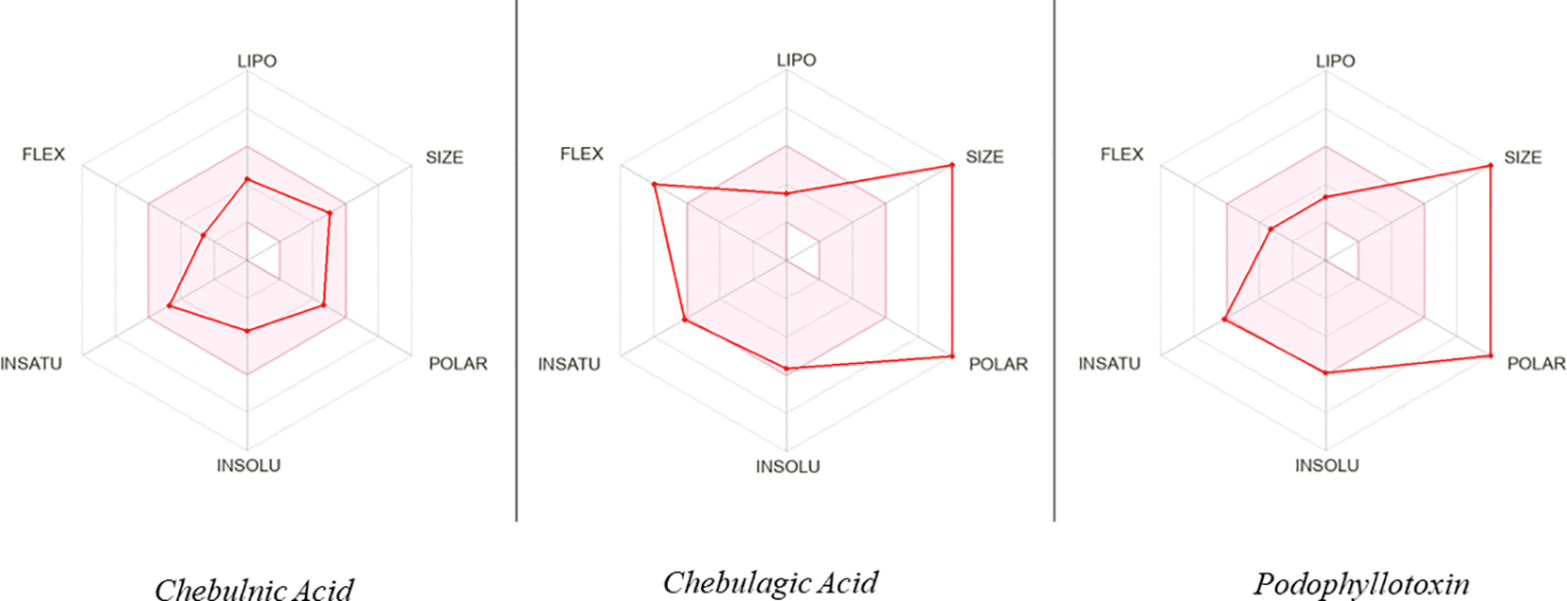
A graphical illustration of the ADMET (Absorption, Distribution, Metabolism, Excretion, and Toxicity) properties of the compounds under investigation.

To assess the potential of the identified phytobioactives as viable therapeutic candidates, we conducted an ADMET analysis. This involved evaluating the compounds for their absorption, distribution, metabolism, excretion, and toxicity (ADMET) profiles. This is important as poor ADMET characteristics can greatly impact the effectiveness of a drug. In silico methods were used to predict the ADMET properties of the compounds and identify any potential issues before moving to the clinical trial phase. Additionally, we also examined the compounds using the AMES toxicity assay to assess their potential toxicity profiles (**Figure 10**, **Table 5**). Overall, the results of our ADMET analysis provide valuable insight into the potential of the screened phytobioactives as therapeutic candidates.

**Table 5:** ADMET identified properties of phytobioactives.

ADMET Entry	Chebulinic acid	Chebulagic acid	Podophyllotoxin
Lipinski	No	No	Yes
Bioavailability Score	0.11	0.11	0.55
Water Solubility	YES	YES	YES
Intestinal Absorption	Low	Low	High
Skin Permeability(cm/s)	-11.67	-11.87	-7.40
P-glycoprotein Substrate	Yes	Yes	No
BBB Permeability	No	No	No
CYP1SA2 Inhibitor	No	No	No
CYP12C19 Inhibitor	No	No	No
CYP2C9 Inhibitor	No	No	No
CYP2D6 Inhibitor	No	No	Yes
CYP3A4 Inhibitor	No	No	Yes
AMES Toxicity	YES	Yes	No

### 3.6 Molecular dynamics simulation results

**Figure 11:**
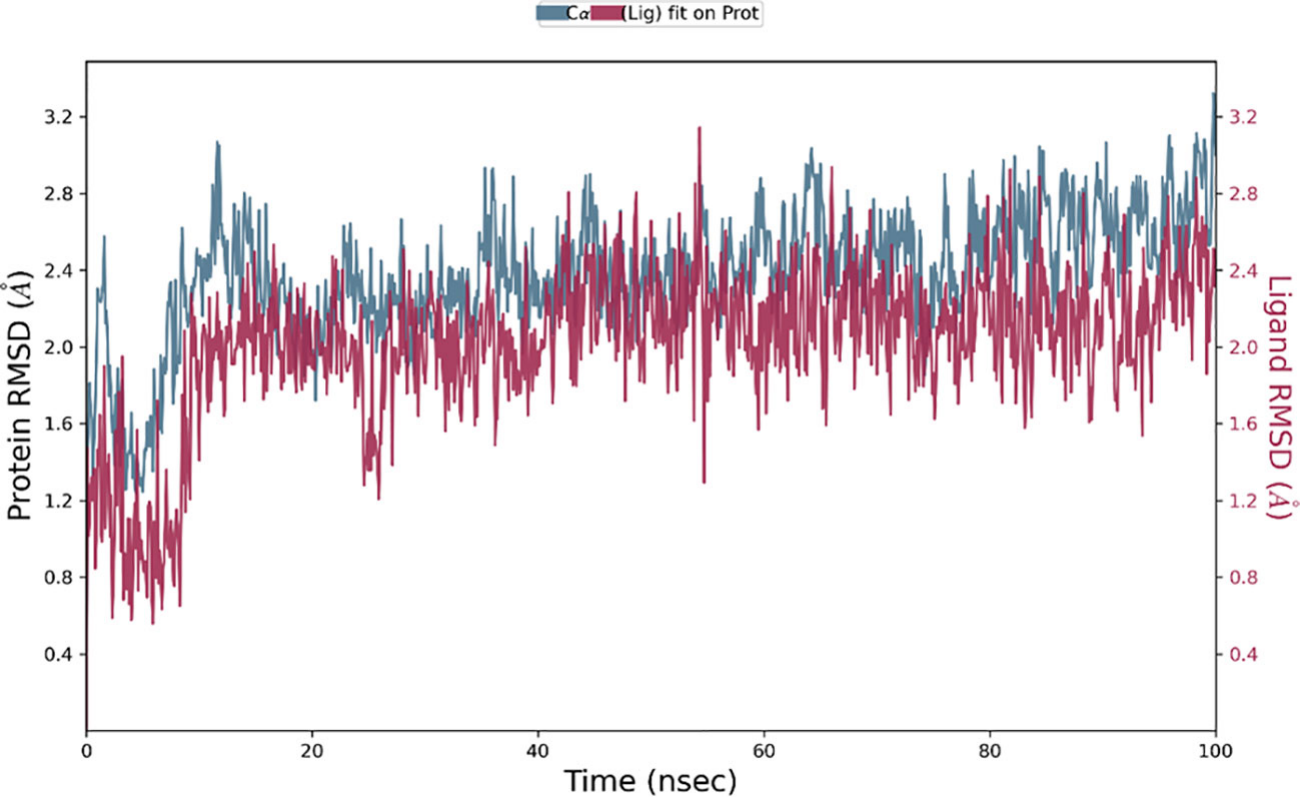
RMSD plot of Chebulagic Acid-FAK complex showing RMSD of Protein backbone(Å) (blue) and Chebulagic acid (Å) (red).

**Figure 12:**
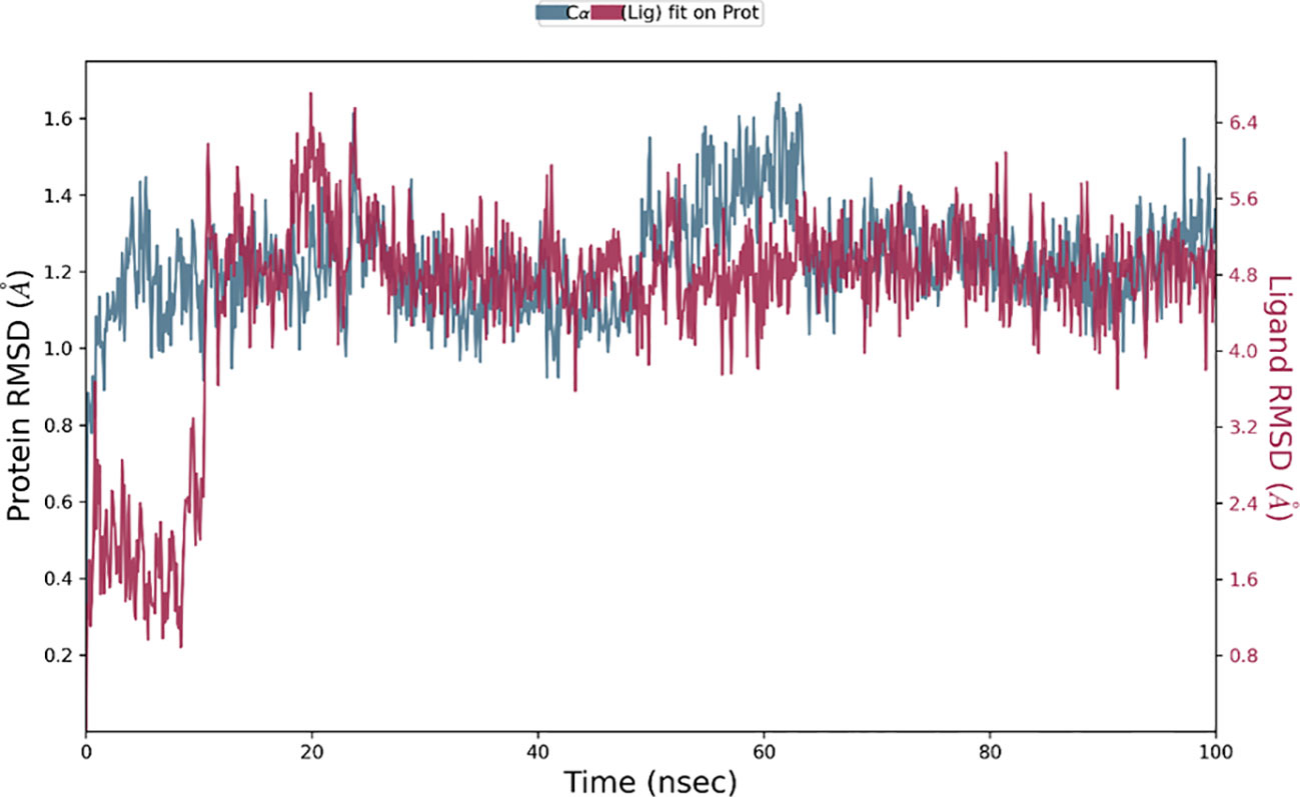
RMSD plot of Chebulinic Acid-VEGF complex showing RMSD of Protein backbone(Å) (blue) and Chebulagic acid (Å) (red).

**Figure 13:**
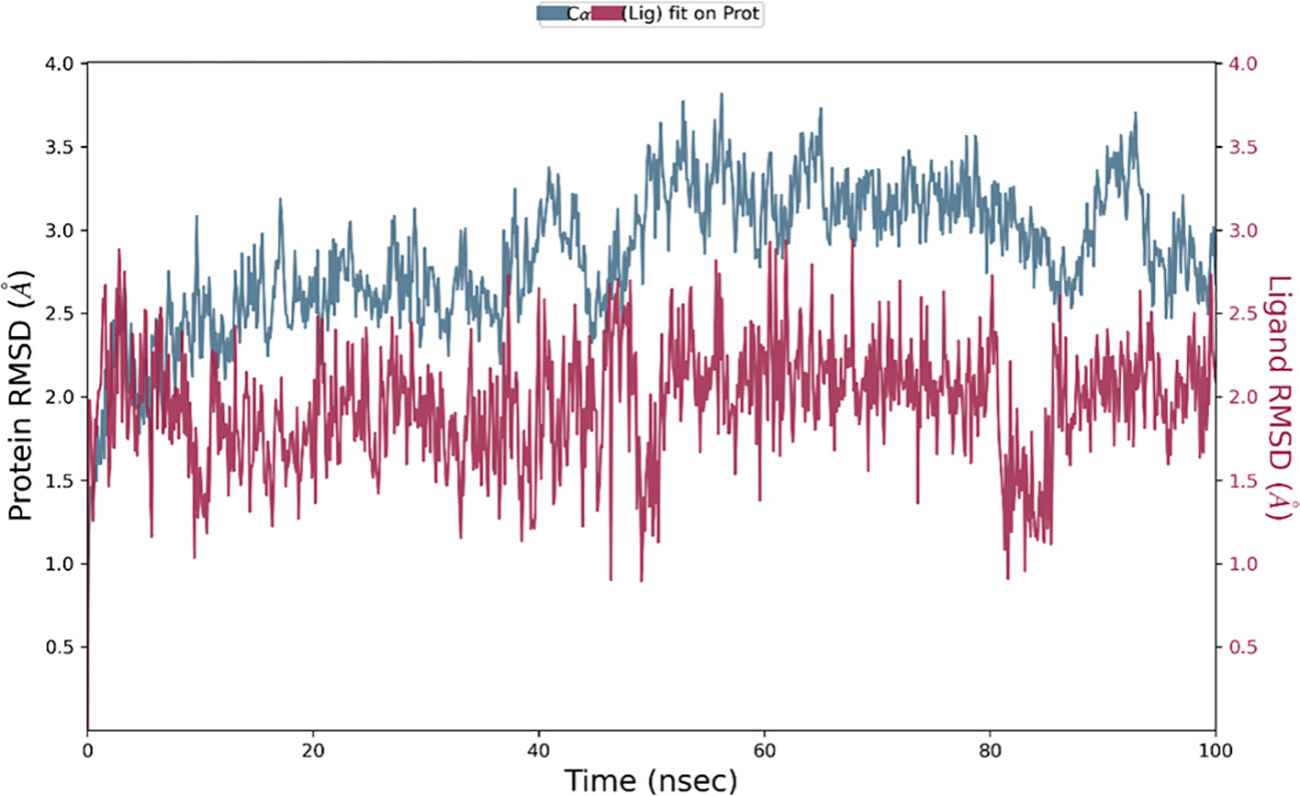
RMSD plot of Chebulinic Acid- MTA1 complex showing RMSD of Protein backbone(Å) (blue) and Chebulagic acid (Å) (red).

The Desmond software was utilized to compute the root-mean-square deviation (RMSD) of the protein-ligand complex during a molecular dynamics (MD) simulation, and the results were graphically shown (**Figures 11**–**13**). The RMSD plot offers valuable information regarding the stability and structural alterations of the protein-ligand complex throughout the simulation. A smaller RMSD value signifies a higher degree of structural similarity between the complex and the reference. The protein RMSD (blue colored plot) indicates the structural conformation throughout the time frame. Based on the results, higher conformational changes were observed for the protein MTA. FAK RMSD stabilized after 10 ns between 2 to 3 Å. Lowest conformational changes were observed for the VEGF RMSD plot with slight fluctuations between 50 to 65 ns. The ligand RMSD determines the stability when docked to the protein binding pocket. Chebulagic Acid complexed with FAK and Chebulinic with VEGF initially fluctuated for 15 ns and later stabilized throughout the run between 1.6 Å to 2.6 Å and 4.4 to 5.8 Å but the latter showed a higher RMSD compared to the other complexes. The Chebulinic showed comparatively more fluctuations throughout the run when complexed with MTA1 varying between 1 to 2.5 Å.

## 4 Discussion

Cancer remains a significant challenge in the healthcare industry, with a high mortality rate that has persisted over the past few decades. Recent studies have identified the crucial roles of Focal Adhesion Kinase (FAK), VEGF and MTA1 in the progression of breast cancer, which has shed new light on this issue. Despite the evolving biological paradigms that regulate tumor proliferation, metastasis and response to therapy, the ability of tumor cells to maintain sustained angiogenesis and to invade and metastasize the surrounding tissues continues to dominate the pathological hallmarks of cancers. Hence, we have attempted to identify bioactive compounds from natural molecules that might potentially inhibit tumor progression without posing the challenge of chemoresistance which is more obvious in synthetic molecules. As witnessed by our computational studies, the remarkable binding affinity of the bioactive compounds of *Terminalia elliptica* namely Chebulinic acid with VEGF, MTA1 and Chebulagic acid with FAK do hint us of the possible inhibitory potential of this natural molecule to combat tumor proliferation and metastasis targeting VEGF mediated angiogenesis, and FAK regulated and MTA1 associated metastases. In this study, we aimed to investigate the potential of *T. elliptica* phytobioactives to regulate FAK protein, VEGF and MTA1.

Terminalia species, which are traditionally used to treat malaria and other infectious diseases, were screened for their anticancer properties. Chebulagic acid, a tannin molecule known for its antibacterial, neuroprotective, radiation-protective, anti-inflammatory, cardio protective, antiviral, antidepressant-like, anti-adipogenic, and antioxidant properties, was studied for its anticancer potential. Using cutting-edge bioinformatics tools and software, we performed an extensive in silico evaluation of 32 screened phytobioactives against FAK and MTA1. The results of the study are highly encouraging, as they showed that the screened phytobioactives from *Terminalia elliptica* displayed remarkable binding interactions and affinity for FAK, VEGF R1, and MTA1 compared to the standard drug podophyllotoxin. Molecular docking and MD simulation analysis showed promising binding stability of the tannin compounds chebulagic acid and chebulinic acid. These results suggest that these compounds could be potential lead molecules for FAK inhibition and a potential MTA1/VEGF inhibitor, paving the way for the development of effective cancer treatment drugs. Overall, our findings suggest that phytobioactives from *Terminalia elliptica* have anti-cancer properties and could be potential therapeutic agents for cancer.

This study provides new insights into the molecular mechanisms underlying the anticancer effects of *Terminalia elliptica* and highlights its potential as a therapeutic agent for cancer treatment. Modulation of FAK, VEGF R1, and MTA1 by phytobioactives may hold promise for slowing the progression of cancer. Nonetheless, further research is necessary to validate these findings and to explore the full potential of these compounds in cancer therapy.

## 5 Conclusion

Our preliminary data relies on the computational methods to identify VEGF, FAK and MTA1 as targets for *Terminalia elliptica* derived Chebulagic acid and Chebulinic acid as probable therapeutic candidates against tumor angiogenesis and metastasis. However, further evidence based experimental studies with cell based, *in situ* and *in vivo* models need to be performed to validate our data to identify the mechanism of action of this molecule in regulating VEGF, FAK and MTA1 mediated signal transduction pathways. However, the above three proteins play a crucial role in different pathways that help in cancer proliferation. Regulation of these proteins can lead to cancer prevention. Nonetheless, the results of this study are encouraging and could lead to the development of more efficient strategies for combating cancer. A detailed study on the toxicity, pharmacokinetics and pharmacodynamics of these molecules will allow us to confirm the safety and efficacy of this molecule for improved therapeutic outcomes. However, this study proposes a novel multi-protein targeted approach of development of drug candidates and provides a foundational basis for further exploration of these molecules for anti-cancer therapy.

## Data Availability

The original contributions presented in the study are included in the article/supplementary material. Further inquiries can be directed to the corresponding authors.
